# Biodegradable Metal Organic Frameworks for Multimodal Imaging and Targeting Theranostics

**DOI:** 10.3390/bios11090299

**Published:** 2021-08-27

**Authors:** Xiangdong Lai, Hui Jiang, Xuemei Wang

**Affiliations:** State Key Laboratory of Bioelectronics (Chien-Shiung Wu Lab), School of Biological Science and Medical Engineering, Southeast University, Nanjing 210096, China; ab58472416@163.com (X.L.); sungi@seu.edu.cn (H.J.)

**Keywords:** biodegradable materials, metal-organic framework, metal ion nodes, multimode imaging, theranostic nano-platforms

## Abstract

Though there already had been notable progress in developing efficient therapeutic strategies for cancers, there still exist many requirements for significant improvement of the safety and efficiency of targeting cancer treatment. Thus, the rational design of a fully biodegradable and synergistic bioimaging and therapy system is of great significance. Metal organic framework (MOF) is an emerging class of coordination materials formed from metal ion/ion clusters nodes and organic ligand linkers. It arouses increasing interest in various areas in recent years. The unique features of adjustable composition, porous and directional structure, high specific surface areas, biocompatibility, and biodegradability make it possible for MOFs to be utilized as nano-drugs or/and nanocarriers for multimodal imaging and therapy. This review outlines recent advances in developing MOFs for multimodal treatment of cancer and discusses the prospects and challenges ahead.

## 1. Introduction

**Figure 1:**
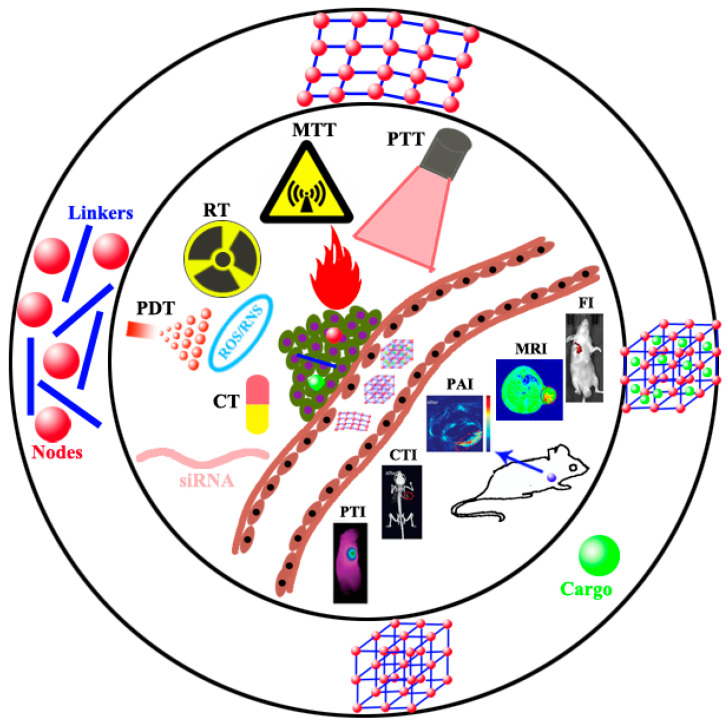
Schematic diagram of MOFs as nano-drugs and nanocarriers for multimodal theranostic, typically comprising a suitable and effective combination of CT, CDT, RT, RDT, PTT, PDT, MDT, MTT, gas therapy and gene therapy, and imaging of FI, MRI, PAI, CTI and PTI.

Cancer has been a serious threat to human health [1]. The accurate therapy of cancer still needs to overcome many great difficulties [2,3]. Each therapy, such as chemotherapy (CT), chemo-dynamic therapy (CDT), radiation therapy (RT), radio-dynamic therapy (RDT), microwave thermal therapy (MTT), microwave dynamic therapy (MDT), photothermal therapy (PTT), and photodynamic therapy (PDT), has its inherent advantages and defects [4,5,6]. Hence, the treatment of cancers has gradually developed from the past monotherapy mode to the current multimode synergistic therapy to enhance therapeutic effects. With the rapid development of nanotechnology, the realization of multimode synergistic therapy depends largely on how to integrate multiple treatment modes into a single nano-platform rather than purely carrying out physical mixing to obtain a simple additive treatment effect. In recent years, emerging and rapidly developing MOF materials have shown enormous potential in multimodal synergistic therapy because of their unique porous structure and characteristics. MOF is a kind of organic inorganic hybrid material through coordination bonds formation between metal ion/ion clusters nodes and organic ligand linkers [7,8,9,10,11]. MOF characterizes by variable compositions and structures, adjustable porosity and pore sizes, high surface areas, good biocompatibility, and biodegradability [7,12,13]. Weak coordination bonds can endow MOFs with a stable but degradable structure [14,15,16]. Nanoscale pores and an ordered crystal structure allow MOFs to accumulate in tumor through enhanced permeability and retention effect (EPR) [17,18]. At the same time, the organic linker can be additionally functionalized for targeting cancer therapy [19,20]. Furthermore, the good dispersibility and biocompatibility of specific MOFs can ensure the biosafety of targeting treatment in vivo [21]. Adjustable composition results in controlled synthesis with different morphology, size, and chemical properties, making MOF itself a nano-drug for multimodal imaging and therapy by choosing appropriate metal nodes and organic ligands [22,23]. Moreover, the porous and ordered structure and the high ratio surface areas are suitable for efficient loading of various cargos for multimodal imaging and therapy [24,25,26,27,28]. MOF-based nanomaterials were applied to fluorescence imaging (FL) [29,30,31,32], photoacoustic imaging (PAI) [33], magnetic resonance imaging (MRI) [34,35,36], computed tomography imaging (CTI) [37,38,39], photothermal imaging (PTI) [40,41] and positron emission tomography (PET) imaging [42,43,44,45]. It is worth mentioning that multimodal imaging [46,47,48,49,50,51,52] is beneficial for tumor diagnosis and accurate position. MOF-based heterogeneous hybridization may serve as an effective methodology for multimodal imaging and synergistic therapy. It integrates the advantages of various materials and endues the hybrid materials with whole new physicochemical properties, realizing theranostic nano-platforms through multimode imaging-guided therapy. In this review, the development of biodegradable MOFs as nano-drugs and nanocarriers for multimodal imaging and therapy in recent years will be summarized and discussed, as shown in Figure 1, and the prospects and challenges of MOFs in multimodal synergistic treatment will also be explored for promising clinic/biomedical applications.

## 2. MOFs as Nano-Drugs

**Figure 2:**
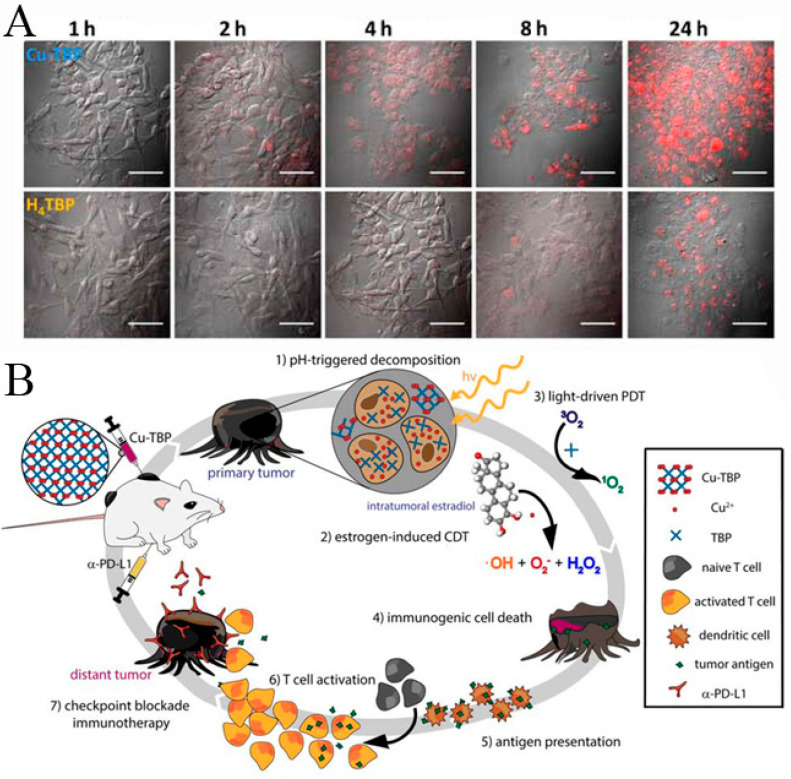
(**A**) B16F10 cellular uptake of Cu-TBP or H_4_TBP at different time-points after incubation with equivalent TBP concentrations of 20 mM observed by confocal imaging. Free H_4_TBP emits red fluorescence. Scale bar, 50 μm. (**B**) Synergy of Cu-TBP mediated radical therapy stimulated by hormone, light and checkpoint blockade immunotherapy. Reprinted with permission from Ref. [54]. Copyright 2019, Elsevier.

Due to the nearly infinite combination of metal ion/ion clusters nodes and organic ligand linkers, the physical and chemical properties of MOFs could be regulated for many applications. Through careful selection and design, metal ion/ion clusters nodes and organic ligand linkers can be directly and fully utilized as nano-drugs to realize multimodal imaging and therapy. Liu et al. [53] reported a nanoscale MOF synthesized by hafnium (Hf^4+^) and tetra (4-carboxyphenyl) porphyrin (TCPP), in which TCPP as a photosensitizer converted tissue oxygen to cytotoxic singlet oxygen under light irradiation and could be used for PDT. At the same time, Hf^4+^ characterized by strong X-ray absorption capacity could act as a radiation sensitizer to enhance RT. Compared to other metals with a higher atomic number, Hf was relatively safe and showed no apparent biological toxicity. Hf-TCPP MOF was biodegradable and easily removed from the mouse body. Hf-TCPP MOF as a biodegradable carrier-free system was used for combined RT and PDT in vitro and in vivo, demonstrating a remarkable anti-tumor effect. Lin’s group [54] reported Cu-TBP (5,10,15,20-tetrabenzoatoporphyrin) nanoscale MOF mediated synergistic hormone-induced CDT and light-induced PDT in the tumor model with high estradiol expression. The degradable Cu-TBP MOFs were accumulated in tumor cells efficiently and decomposed into Cu^2+^ and H_4_TBP by monitoring free porphyrin fluorescence, which was entirely quenched by the paramagnetic Cu^2+^ in intact MOF at pH 7.4 and reappeared in acid tumor cell microenvironment due to the decomposition of Cu-TBP (Figure 2A). Cu-TBP was injected into dorsal subcutaneous tumors and produced Cu^2+^ and porphyrin in the low pH tumor microenvironment. Cu^2+^ ions, as redox-active metal centers, catalyzed estradiol metabolism to generate hydrogen peroxide, hydroxyl radical (·OH), superoxide (O^2−^) species, and other ROS for CDT, whereas H_4_TBP mediated light-induced PDT. This MOF-mediated radical treatment depleted intratumoral estradiol and inhibited tumor growth. Upon light irradiation, H_4_TBP produced ROS to destroy the irradiated cancer cells, causing immunogenic cell death and tumor antigens release. Released tumor antigens and injected PD-L1 antibody caused the effective T cell proliferation and infiltration into the tumor, overcoming the immunosuppressive tumor microenvironment and simultaneously effectively inhibiting the growth of distant tumors (Figure 2B).

**Figure 3:**
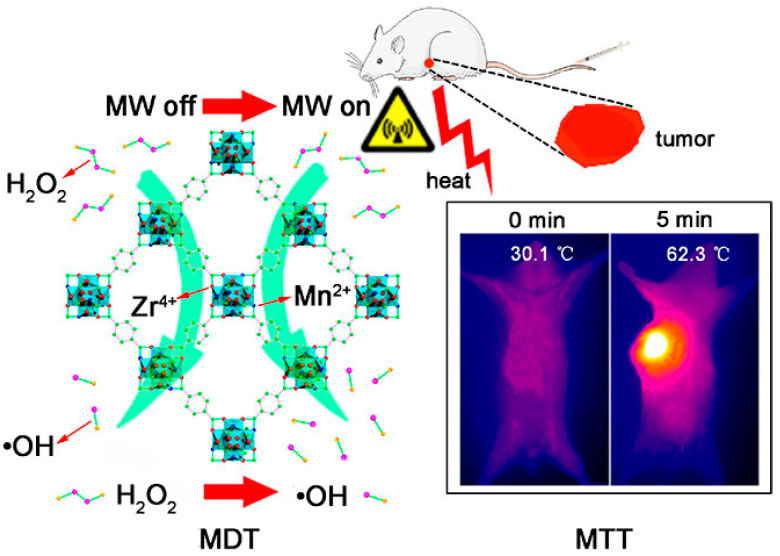
Mn-Zr MOF generates abundant ROS of ·OH and a high microwave thermal conversion efficiency after exposure to MW irradiation, resulting in efficiently inhibiting the cancerous cell growth through the synergic effect of MDT and MTT. Reprinted with permission from Ref. [55]. Copyright 2018, American Chemical Society.

Zirconium(IV) chloride (ZrCl_4_), Manganese(II) chloride tetrahydrate, and 1,4-Benzenedicarboxylic acid (H_2_BDC) were used as raw materials to chemically synthesize Mn-doped Zr MOF by a one-pot hydrothermal method [55]. The flexible and microporous structure is beneficial to the strongly confined inelastic collision of ions, resulting in a significant microwave thermal conversion efficiency as high as 28.7%. The Mn-ZrMOF catalyzed the degradation of H_2_O_2_ to generate ·OH under MW irradiation. The in vitro and in vivo experimental studies confirmed that a union of MTT and MDT with simultaneous generation of heat and ROS under mild MW irradiation realized synergistic inhibition of the growth of tumors, as schematically reported in Figure 3. The Mn-ZrMOF was degradable in vivo and excreted out of the body gradually, demonstrating that it is a bio-safe therapeutic nano-agent.

**Figure 4:**
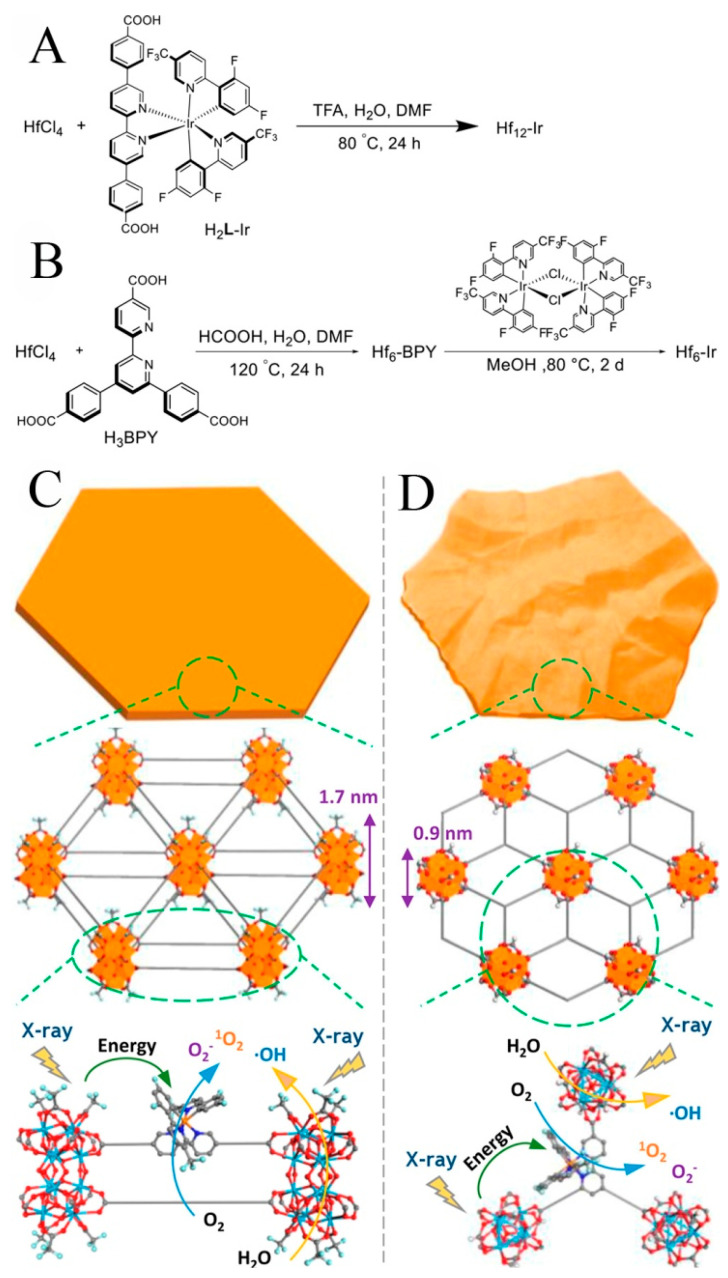
The synthesis methods, morphologies and structures of Hf_12_-Ir MOF nanolayer (**A**,**C**) and Hf_6_-Ir MOF nanolayer (**B**,**D**) and X-ray induced ROS generation. Reprinted with permission from Ref. [56]. Copyright 2018, American Chemical Society.

Lan et al. [56] reported two MOF nanolayers, Hf_12_-Ir, and Hf_6_-Ir (namely Iridium), schematically reported in Figure 4. Under X-ray irradiation, electron-dense Hf_12_ and Hf_6_ secondary building units not only generated ·OH to enhance RT but also transfered energy to photosensitizing Ir (2,2′-bipyridine) [2-(2,4-difluorophenyl)-5-(trifluoromethyl) pyridine]_2_^+^ to generate single oxygen (^1^O_2_) and O_2_^−^, resulting in RDT. RT and RDT exerted superb anticancer effects at shallow X-ray doses.

**Figure 5:**
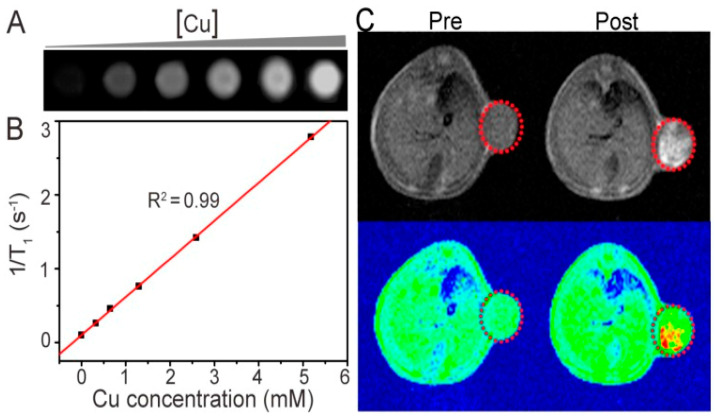
(**A**) MRI of the Cu-TCPP aqueous solution with different concentrations. (**B**) Plots of the 1/T1 value of the Cu-TCPP under concentration dependence. (**C**) mouse MRI before and after intratumoral injection of the Cu-TCPP. Red circles indicate the position of the tumor. Reprinted with permission from Ref. [41]. Copyright 2018, Ivyspring International Publisher.

Wu et al. prepared Cu-TCPP MOF nanosheets for dual-modal PTT and PDT. Upon 808 nm laser irradiation, the coexisting Cu^+^ and Cu^2+^ exhibited excellent photothermal performance due to the strong near-infrared (NIR) absorption [41]. In the meantime, TCPP produced ^1^O_2_ for PDT. The toxicity experiment indicated that Cu-TCPP has good biocompatibility. Due to Cu(II) in the Cu-TCPP nanosheets, near-infrared thermal imaging and T1-weighted magnetic resonance imaging (MRI) could be used to realize simultaneous diagnosis and therapy (Figure 5).

## 3. MOFs as Nanocarriers

**Figure 6:**
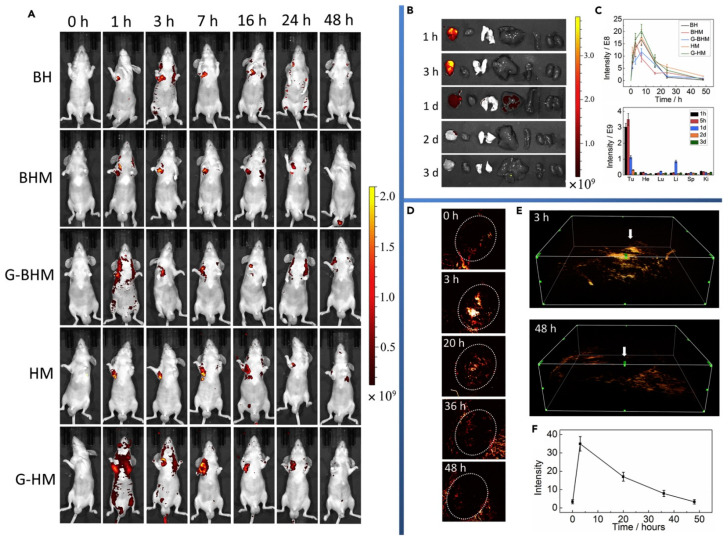
(**A**) Fluorescence imaging of SGC-7901 tumor-bearing model mice after intravenous injection of different materials at 7 time points. Unit of scale bar: (p/s/cm^2^/sr)/(mW/cm^2^). (**B**) Ex vivo Fluorescence imaging of tumor, heart, lung, liver, spleen, and kidney in sequence in SGC-7901 tumor-bearing model mice after intravenous injection of G-BHM at 5 different time points. (**C**) Time-dependent in vivo integrated FL intensity for different materials (top) and in different organs (bottom). (**D**) PA imaging of SGC-7901 tumor after injection of G-BHM at different time points. (**E**) Stereoscopic PA images, and white arrow represents tumor zone. (**F**) PA signal intensity variation corresponding to part (**D**). Reprinted with permission from Ref. [66]. Copyright 2019, Elsevier Inc.

The porous and ordered structure, tunable sizes, and the high ratio surface areas make MOF easy to be loaded a variety of cargos efficiently and increase the cargo capacity. In 2006, Patricia first reported a MOF for drug delivery named Materials of Institute Lavoisier 100 and 101 (MIL-100 and 101) [57]. After more than ten years of rapid development, MOF was used to carry oxygen [58], chemotherapeutic agents [59], photosensitizer [60], photothermal conversion agents [15,61], Nucleic acids and proteins [62,63,64,65]. Du et al. [66] reported an intelligent stimuli-responsive and completely degradable MOF delivery system. Based on a ‘‘framework exchange’’ strategy, black phosphorous quantum dots (BPQDs) were embedded into ZIF-8 nanoparticles, which were used as sacrificial templates to prepare BP@HKUST-1 (BH). MIL-100(Fe) shell enveloped the BH core to form the core-shell structure, while s-nitroso-glutathione was encapsulated into HKUST-1@MIL-100. In tumor cells, the high levels of glutathione and ROS triggered the decomposition of s-nitroso-glutathione to produce NO and ·OH, causing the damage of mitochondria and DNA in tumor cells. Black phosphorus has superb biocompatibility and very high photothermal conversion efficiency. This MOF was fluorescent and photoacoustically active (Figure 6), allowing it to readily achieve accurate multiple therapies that use gas, free radicals, and PTT. Notably, this nanosystem completely degraded into phosphate radicals, terephthalic acid, and metal ions excreted out of the body.

**Figure 7:**
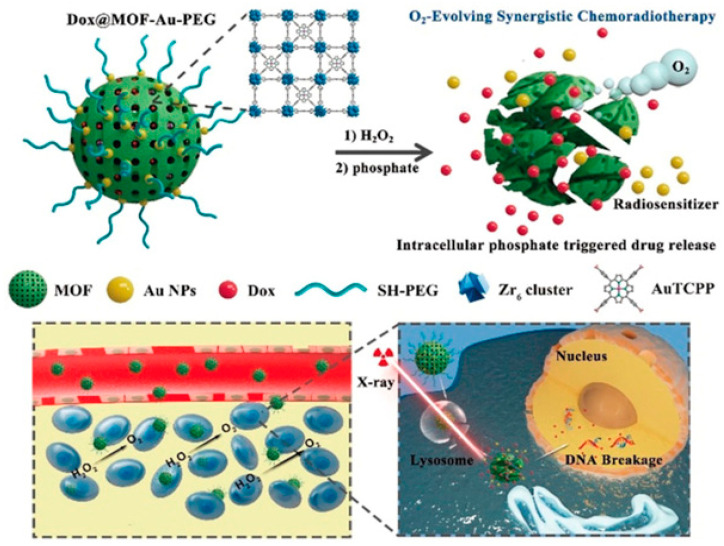
The structure of Dox@MOF-Au-PEG and the underlying of O_2_-generating synergistic chemoradiotherapy Reprinted with permission from Ref. [67]. Copyright 2019, Wiley-VCH Verlag GmbH & Co. KGaA, Weinheim.

He et al. fabricated a MOF consisting of Zr^6+^ nodes and TCPP ligand [67]. The gold nanoparticles (AuNPs) were decorated on the surface of MOF, which was conducive to effectively stabilize the nanostructure and increased radiotherapy sensitivity. Meanwhile, chemotherapeutic drug doxorubicin (Dox) was encapsulated into the MOF. The fabricated MOFs were densely packed polyethylene glycol (PEG) corona to form Dox@MOF-Au-PEG. Dox@MOF-Au-PEG oxygenated tumor microenvironment by catalyzing the degradation of H_2_O_2_ in tumor into O_2_, resulting in enhancing O_2_-dependent radiotherapy. Dox@MOF-Au-PEG combined the radiotherapy sensitization effect of AuNPs and the anticancer effect of Dox, achieving synergistic chemoradiotherapy, as shown in Figure 7. The stronger coordination interaction between phosphate ion and zirconium made the MOF readily decompose in PBS (2 mM), resulting in the burst release of porphyrin ligands and structural collapse. Once MOF was internalized by cancer cells, the phosphate in high concentration led to the disassembly of the NPs.

**Figure 8:**
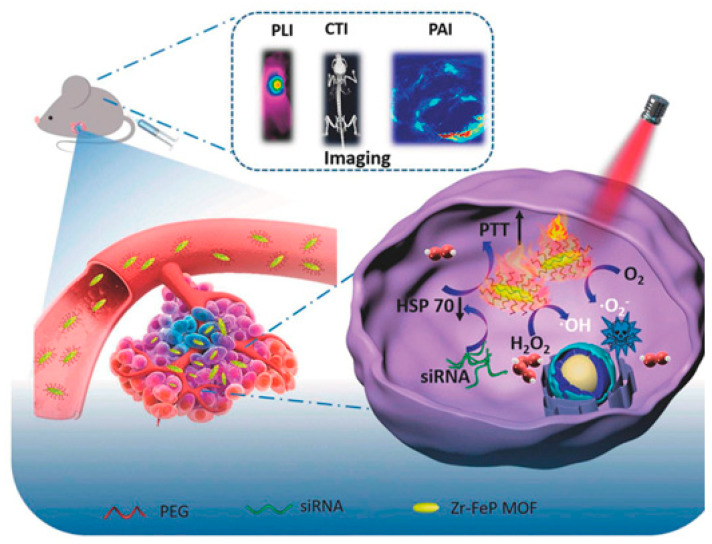
siRNA/Zr-FeP MOF mediates PTT at a lower temperature and PDT for cancer. Reprinted with permission from Ref. [46]. Copyright 2018, Elsevier B.V.

Zhang et al. [46] prepared a porous zirconium-ferriporphyrin MOF nano-shuttles (Zr-FeP) made from ZrOCl_2_∙8H_2_O and H_4_TBP-Fe, carrying the siRNA of 70 kDa heat shock protein (HSP70). Under NIR lasers, the siRNA/Zr-FeP MOF catalyzed endogenous H_2_O_2_ and O_2_ to become ·OH and ^1^O_2_, while it had high photothermal conversion efficiency up to around 34%. Moreover, siRNA reversed the HSP70-mediated thermotherapy resistance, achieving PTT at a lower-temperature and avoiding the nearby normal tissues from the nonspecific thermal radiation damage. The siRNA/Zr-FeP significantly suppressed the tumor cell growth in vitro and in vivo through the synergistic effect of PTT at a lower temperature and PDT, shown in Figure 8. siRNA/Zr-FeP was effectively cleared out of the organism, via its gradual decomposition into small molecules and ions. Meanwhile, MOF nano-shuttles achieved PAI, CTI, and photothermal imaging (PTI) tri-mode tumor-specific imaging capability, providing a powerful theranostic tool for tumors.

**Figure 9:**
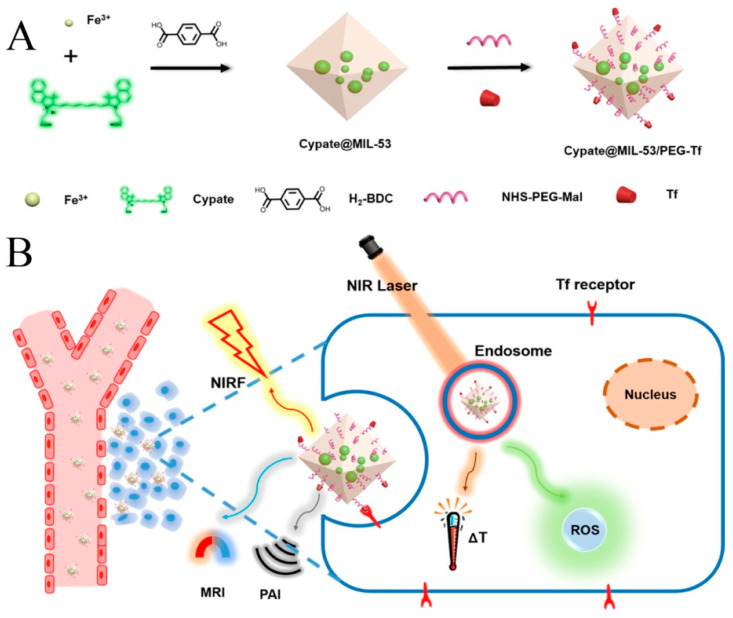
(**A**) the preparation of Cypate@MIL-53/PEG-Transferrin MOF composite and (**B**) its bioapplication for PDT and PTT. Reprinted with permission from Ref. [70]. Copyright 2019, American Chemical Society.

Liu et al. [68] encapsulated BPQDs and catalase into MIL-101 inner and outer layers, respectively, and constructed a MOF heterostructure, BPQDs-MIL@catalase-MIL. BPQDs exhibited two abilities of photothermal conversion for PTT and ^1^O_2_ production for PDT. The catalase in the outer layer catalyzed H_2_O_2_ into O_2_. O_2_ was then converted into ^1^O_2_ by BPQDs in the inner layer. The PDT/PTT synergistic therapy accelerated cancer cell apoptosis. Ni et al. [69] reported a Hf-DBB-Ru MOF consisting of Hf^4+^ and bis(2,2′-bipyridine) [5,5′-di(4-benzoate)-2,2′-bipyridine] ruthenium chloride for mitochondrial-targeted RDT and RT. Ru endowed Hf-DBB-Ru with strong mitochondria-targeted ability. Hf clusters generated abundant ·OH, and Ru-based linkers produced ^1^O_2_ at low dose X-ray irradiation with high penetration. Yang et al. [70] developed cypate@MIL-53 nanoparticles. Fe^3+^ metal ions and the carboxyl group of cypate interacted to form precursor complexes, improving bioavailability and protecting the cypate NIR dye from photobleaching. Organic linkers H_2_BDC coordinated with Fe^3+^ to generate crystallized MOFs. PEG and transferrin were functionalized on the surface of cypate@MIL-53 to enhance biocompatibility and tumor targeted functions. Cypate molecules gave this MOF the ability to behave as photosensitizers and photothermal agents for PDT and PTT, as shown in Figure 9. This MOF realized tumor targeted multimodal imaging (Near-infrared fluorescence images, PAI, and MRI).

**Figure 10:**
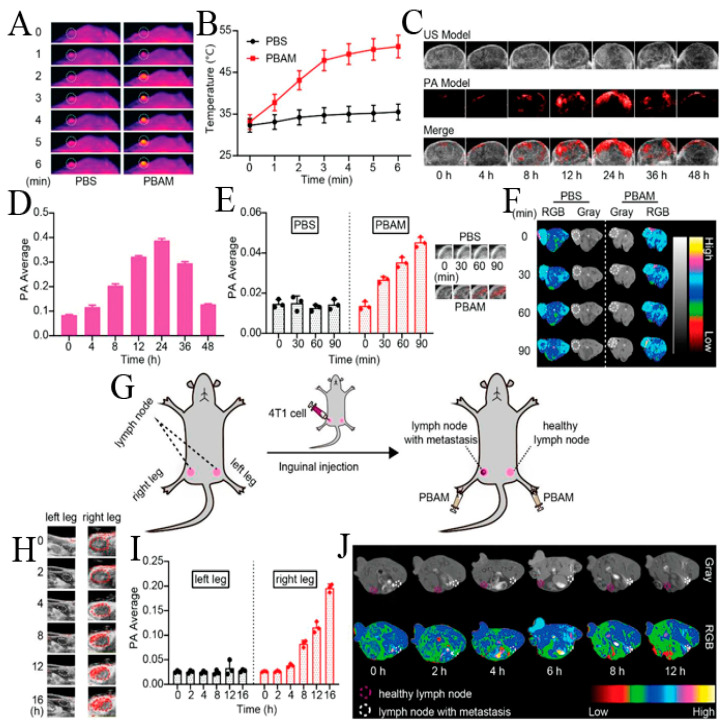
(**A**) Infrared thermal imaging and (**B**) tumor temperature of 4T1-tumor model mice after intravenous injection with PBAM under 808 nm laser (1 W cm^−2^). White circle: tumor tissue. (**C**) Tumor PAI and (**D**) Corresponding PAI signal intensity of 4T1-tumor model mice after intravenous injection with PBAM. (**E**) Tumor PA signal intensity and corresponding PAI of 4T1-tumor model mice after subcutaneous injection with PBAM. (**F**) MRI of 4T1-tumor model mice after subcutaneous injection with PBAM. White circle: tumor tissue. (**G**) Schematic of lymphatic metastasis tumor model. (**H**) PAI of lymph nodes with or without metastasis at different time points after injection with PBAM. White circle: the lymph nodes in left leg. Red circle: the lymph nodes in right leg. (**I**) Corresponding PAI signal intensity of lymph nodes with or without metastasis. (**J**) MRI of lymph nodes with or without metastasis at different time points after injection with PBAM. Reprinted with permission from Ref. [20]. Copyright 2020, WILEY-VCH Verlag GmbH & Co. KGaA, Weinheim.

Chen et al. synthesized MIL-100 (Fe) coated Mn-based Prussian blue (PB) analogue (K_2_Mn[Fe(CN)_6_]), named as PBAM, by simply stirring and heating, losing photothermal activity of PB and T1-weighted MRI due to local confinement of Mn^2+^ [20]. In the mildly acidic tumor microenvironment, the MIL-100 shell was degraded, and the released Fe^3+^ exchanged with Mn^2+^ to synthesize in situ the more stable PB, Fe^3+^–[Fe(CN)6]^4−^, and to release free Mn^2+^. Mn^2+^ reacted with endogenous H_2_O_2_ and HCO^3−^ and generated ·OH for CDT. The excellent PAI and PTT of PB, and T1-weighted MRI and CDT of Mn^2+^ showed accurate theranostic effects (Figure 10).

## 4. Summary and Perspectives

Weak coordination bonds result in degradable structures, which is very good for biomedical applications of MOFs. Furthermore, it is of great significance to develop MOFs as nano-drugs for multimodal imaging and therapy. MOFs itself as nano-drugs characterize by simplicity and efficiency, high drug loading, and lower dosages of node and linker drugs, benefiting for achieving expected anti-tumor effects and reducing toxic effects on normal tissues and cells. MOF specific features, such as flexible and diversified morphologies, tunable sizes, high surface areas, and tunable pore diameter make MOFs intelligent nanocarriers for multimodal diagnosis and therapy, easy to be loaded with a variety of cargos efficiently and harbor increased cargo capacity. To sum up, we have reviewed in detail the recent progress of biodegradable MOFs for multimodal theranostic, typically comprising a suitable and effective combination of CT, CDT, RT, RDT, PTT, PDT, MDT, MTT, gas therapy, and gene therapy, and imaging of FI, MRI, PAI, CTI, and PTI.

The efficacy of single modal therapy is often not ideal due to multidrug resistance, nonspecific heating, hypoxia, and other serious adverse effects. Though MOFs for multimodal therapy have demonstrated synergistic and enhanced therapeutic efficacy and low cytotoxicity in laboratory research, there is still great room for improvements to realize targeted cancer therapy. Firstly, most excellent researches lacked long-term experiments on the toxicity of MOFs. Comprehensive studies on the absorption, biodistribution, metabolism, excretion, clearance, and long-term tissue accumulation of MOFs are necessary for determining toxicity in vivo. Toxicity is effectively prevented through choosing highly biocompatible metal ions as nodes (e.g., Ca, Fe, Zn, etc.) and endogenous bioactive molecules as ligands [71,72]. The additional loaded cargo needs to be considered as well because of probable threats to the organism. In addition, there is an ever growing need for extensive and in-depth research on the mechanisms and pathways of MOFs degradation in vivo. This is due to the fact that current single imaging methods are not sufficient to monitor and recognize the degradation process of MOFs. Finally, current studies mainly focused on dual-modal therapy [73,74,75,76,77], while very few reports on tri- or more modal therapies were based on MOFs, demonstrating a more effective therapy. In brief, despite facing these challenges, a significant effort has been made to develop biodegradable MOFs for multimodal imaging and therapy, which can realize clinical translations and other bio-applications in the future.

## Data Availability

Not applicable.
